# Postoperative complications influencing the long‐term outcome of head‐injured patients after decompressive craniectomy

**DOI:** 10.1002/brb3.1179

**Published:** 2018-12-04

**Authors:** Guangfu Di, Yuhai Zhang, Hua Liu, Xiaochun Jiang, Yong Liu, Kun Yang, Jiu Chen, Hongyi Liu

**Affiliations:** ^1^ Department of Neurosurgery The Affiliated Brain Hospital with Nanjing Medical University Fourth Clinical College of Nanjing Medical University Nanjing China; ^2^ Department of Neurosurgery First Affiliated Hospital of Wannan Medical College Wuhu China; ^3^ Institute of Neuropsychiatry, The Affiliated Brain Hospital with Nanjing Medical University Fourth Clinical College of Nanjing Medical University Nanjing China; ^4^ Institute of Brain Functional Imaging Nanjing Medical University Nanjing China

**Keywords:** decompressive craniectomy, outcome, postoperative complications, risk factor, traumatic brain injury

## Abstract

**Objective:**

Postoperative complications are common in patients who underwent decompressive craniectomy (DC) after traumatic brain injury (TBI). However, little is known about the degree of association between the postoperative complications and the long‐term outcome of adult TBI patients after DC. The aim of this study was to evaluate the risk of postoperative complications that influenced the long‐term outcome of DC in TBI patients.

**Method:**

A total of 121 patients were studied up to 6 months after DC in TBI. The collected data included demographic, clinical and radiological information, postoperative complications, and Glasgow Outcome Scale‐Extended (GOSE) scores at follow‐up 6 months after DC. Based on their GOSE scores, they were divided into two functional groups: favorable (GOSE = 5–8) or unfavorable outcome (GOSE = 2–4) group. The characteristics of the two groups were compared using statistical analysis. Finally, a regression model was established and a receiver operating characteristic (ROC) curve was applied to analyze its performance power.

**Results:**

Of 121 admitted patients, 31 (25.62%) sustained an unfavorable outcome. A logistic regression analysis showed that the presence of Glasgow Coma Scale (GCS) scores on admission (odds ratio [OR] 0.285, *p* = 0.001), posttraumatic hydrocephalus (PTH) (OR 8.688, *p* = 0.003), craniectomy site (OR 8.068, *p* = 0.033), and postoperative progressive hemorrhagic injury (PHI) (OR 6.196, *p* = 0.026) were independent risk factors that correlated with an unfavorable outcome. Analysis using ROC curves demonstrated that these factors had different accuracies in predicting an unfavorable outcome (AUC = 0.852 for GCS scores on admission; AUC = 0.826 for PTH, AUC = 0.617 for craniectomy site; AUC = 0.616 for postoperative PHI). The performance power of the GCS scores on admission and PTH influenced the patient's outcomes to a similar degree (*p* = 0.623), and either predicted the outcome better than the craniectomy site or the postoperative PHI (*p* < 0.05, respectively).

**Conclusion:**

These findings suggest that the occurrence of PTH and postoperative PHI were independently associated with an unfavorable long‐term outcome after DC in patients with TBI. Early prevention and treatment of PTH and postoperative PHI may be beneficial to improve the long‐term outcome, especially in patients with lower admission GCS scores or bilateral DC.

## 1 INTRODUCTION

Decompressive craniectomy (DC) is a lifesaving procedure used to treat refractory intracranial hypertension that can occur after severe traumatic brain injury (TBI), but despite high survival rates, many patients remain severely disabled (Hutchinson et al., 2016). The loss of human potential and the long‐term impairments and disabilities associated with TBI have a tremendous impact on the family and on society as a whole. In view of these considerable healthcare issues, significant efforts have gone into research to develop and refine management strategies that can improve the outcome following traumatic brain injury (Ho, Honeybul, & Litton, 2011). The outcome of TBI after DC is associated with the severity of the primary brain injury and the effectiveness of preventing any secondary brain injury or postoperative complications (Khalili, Sadraei, Niakan, Ghaffarpasand, & Sadraei, 2016). Primary brain injury in severe TBI occurs at the moment of the mechanical insult and cannot be reversed by surgical intervention (Honeybul & Ho, 2016), but the secondary brain injury and postoperative complications can be prevented by early intervention. Therefore, early diagnosis and treatment of secondary brain injury and postoperative complications are extremely important.

Increased intracranial pressure (ICP) can be induced by many intracranial pathologies caused by TBI and can lead to secondary brain damage (Muzevic & Splavski, 2013). Thus, controlling the ICP is essential in the management of patients with severe TBI. DC is emerging as an effective surgical measure for reducing the ICP and is now considered as a management option for medically refractory ICP (Zhang et al., 2017). However, the complications associated with DC may reduce its potential benefit. Some postoperative complications can be directly fatal, such as postoperative meningitis or progressive hemorrhagic injury (PHI) (Yang et al., 2008). To sustain interest in DC for the management of TBI, a better understanding of the relationship between postoperative complications and the long‐term outcome is required in order to optimize management strategies.

The aim of this study was to evaluate risk factors for postoperative complications that were associated with the long‐term outcome of TBI patients after DC in order to reduce their possible negative impact and to improve the outcome of these patients.

## 2 METHODS

### 2.1 Patient population

Due to the retrospective nature of the study, informed consent was waived; the study was approved by the Institutional Ethical Board of the Yijishan Hospital of Wannan Medical College. Consent for the use of medical records (demographic, clinical and radiological information, postoperative complications and neurological outcome at follow‐up 6 months after DC) was available from all TBI patients who underwent DC between January 1, 2013, and December 30, 2016, at our institution. DC was performed at admission or at a delayed time point due to the presence of a midline shift, contusions, cerebral edema, or intractable intracranial pressure. The inclusion criteria were as follows: (a) age of ≥16 years and presentation of isolated TBI that required unilateral or bilateral DC; and (b) survival of the patients for more than 6 months to allow for follow‐up observations. To avoid interfering factors, patients with a history of neurological disease or intracranial pathological changes before their injury, as well as patients with incomplete medical records were excluded from the study. Finally, 121 patients with moderate or severe TBI after DC were enrolled to determine the risk factors for the development of unfavorable outcomes.

### 2.2 Definition

In this study, postoperative subdural hygroma was defined as a low‐density collection >1 cm in maximal depth measured from the cortical surface to the inner aspect of the scalp (Honeybul & Ho, 2014). Posttraumatic hydrocephalus (PTH) was defined by radiological evidence of ventricular dilatation, determined by an Evans index (or ratio) of >0.3 at the 6‐months follow‐up (De Bonis, Pompucci, Mangiola, Rigante, & Anile, 2010). Postoperative PHI was diagnosed if the postoperative computed tomography (CT) scans showed the presence of new intracerebral hematomas, coalescence of preexisting contusions into a hematoma, delayed or enlarged subdural or epidural hematomas or the development of unexpected postsurgical hematomas at the operative site (Vedantam, Yamal, Rubin, Robertson, & Gopinath, 2016). Posttraumatic epilepsy was defined as the occurrence of one or more seizures occurring late (at least 1 week) after TBI that required treatment with antiepileptic agents (Yang et al., 2008). Posttraumatic cerebral infarction (PTCI) was defined as low‐attenuation lesions in well‐defined arterial vascular distribution on any brain CT scan within 2 weeks after the accident, and a diagnosis of cerebral infarction was revised if follow‐up studies indicated the findings were actually related to evolving contusions, artifacts, or were inconsistently visualized (Ham et al., 2011).

### 2.3 Clinical outcome

The variables and results were evaluated according to the Glasgow Outcome Scale‐Extended (GOSE) at 6 months after injury and a dichotomous variable with favorable outcome (GOSE = 5–8) and unfavorable outcome (GOSE = 2–4) was created (Wilson, Pettigrew, & Teasdale, 1998).

### 2.4 Statistical analysis

Data were analyzed using SPSS (SPSS, Chicago) software. The chi‐square test was employed to identify differences in categorical outcome variables between the favorable outcome group and the unfavorable outcome group. An independent sample *t* test was used to identify the differences between the parametric continuous variables, and the Mann‐Whitney *U* test was used to compare differences in the nonparametric continuous variables between the two groups.

Univariate analysis was performed to explore the differences among all factors between the two groups. Subsequently, binary logistic regression analysis was used to identify independent risk factors for unfavorable outcomes based on the results from univariate analysis. We further evaluated the power of these independent risk factors to discriminate favorable outcome subjects from unfavorable outcome subjects by using receiver operating characteristic (ROC) curves. The statistical threshold was set at *p* < 0.05.

## 3 RESULTS

Of the 121 patients with TBI who underwent DC and that met the inclusion criteria, 85 (70.25%) were males, and the mean age of the patients was 47.79 ± 13.43 years. The major causes of TBI were traffic accidents (62.81%) and falls (33.88%). Of the 121 patients with DC, 103 (85.12%) underwent unilateral DC and 18 (14.87%) underwent bilateral DC. Seventy (57.85%) patients underwent DC within 6 hr after injury, 31 (25.62%) within 6–24 hr, but the majority of patients underwent DC within 24 hr after injury (83.47%). At 6‐month follow‐up, 25.62% of patients had an unfavorable outcome. The clinical information of the 121 patients included in this study is summarized in Table 1.

**Table 1:** Characteristics of 121 patients who underwent decompressive craniectomy procedures after traumatic brain injury

Characteristics	Total (121)	Outcome at 6 months		*p* value
		Favorable (90)	Unfavorable(31)	
Mean age in years (*SD*)	47.79 ± 13.43	46.69 ± 13.66	51.00 ± 12.38	0.124
Sex				
Female	36	28	8	0.654
Male	85	62	23	
GCS scores on admission (*SD*)	6.62 ± 2.02	7.22 ± 1.90	4.87 ± 1.18	＜0.001
Cause of injury				
Traffic accident	76	55	21	0.853
Falling	41	32	9	
Others	4	3	1	
Hypoxemia				
Yes	15	6	9	0.003
No	106	84	22	
Pupil reactivity				
None	29	13	16	＜0.001
One reactive	17	13	4	
Both reactive	75	64	11	
tSAH				
Yes	112	81	31	0.110
No	9	9	0	
Subdural hemorrhage				
Yes	98	71	27	0.429
No	23	19	4	
Epidural hemorrhage				
Yes	27	22	5	0.455
No	94	68	26	
Contusion‐associated hemorrhage				
Yes	103	74	29	0.154
No	18	16	2	
Status of basal cistern				
Absent	49	26	23	＜0.001
Compressed	72	64	8	
Midline shift (cm)	0.82 ± 0.53	0.78 ± 0.50	0.93 ± 0.60	0.193
Craniectomy site				
Unilateral	103	82	21	0.003
Bilateral	18	8	10	
Decompression time (hr)				
＜6 hr	70	47	23	0.088
6 to＜24 hr	31	27	4	
≥24 hr	20	16	4	
Postoperative PHI				
Yes	26	14	12	0.011
No	95	76	19	
Posttraumatic cerebral infarction				
Yes	13	4	9	0.001
No	108	86	22	
Posttraumatic hydrocephalus				
Yes	35	11	24	＜0.001
No	86	79	7	
Postoperative meningitis				
Yes	2	0	2	0.064
No	119	90	29	
Posttraumatic seizures				
Yes	10	5	5	0.122
No	111	85	26	
Subdural hygroma				
Yes	76	54	22	0.293
No	45	6	39	

GCS: Glasgow Coma Scale; PHI: progressive hemorrhagic injury.

The postoperative complications of DC are shown in Table 2. The most frequent complication after surgery was the formation of a subdural hygroma in 76 patients (62.81%). The average time for the occurrence of the subdural hygroma after DC was 9.17 ± 6.52 days. Most subdural hygromas resolved spontaneously; however, in five cases cranioplasty and in nine cases a ventriculoperitoneal shunt helped to resolve the hygroma. Of the 121 patients, 35 patients (28.93%) sustained PTH. The average time for the occurrence of PTH after DC was 58.71 ± 34.49 days, and 23/35 (65.71%) underwent placement of a ventriculoperitoneal shunt. The postoperative CT of 26 (21.49%) patients showed a PHI, and 11/26 (42.31%) required reoperation. Thirteen patients (10.74%) developed PTCI following DC, with the most common site for infarction being in the posterior cerebral artery territory. In ten patients (8.26%), the incidence of posttraumatic seizures required medical treatment. Two patients (1.65%) suffered postoperative meningitis; both of them had an unfavorable outcome at the 6‐months follow‐up.

**Table 2:** Postoperative complications of 121 patients who underwent decompressive craniectomy after traumatic brain injury during 6 months follow‐up

Postoperative complications	*n* (% of total)
Postoperative progressive hemorrhagic injury	26/121 (21.49%)
Epidural hematoma	7
Intraventricular hemorrhage	19
Posttraumatic cerebral infarction	13/121 (10.74%)
Anterior cerebral artery	1
Middle cerebral artery	4
Posterior cerebral artery	8
Subdural hygroma (SDG)	76/121 (62.81%)
Ipsilateral SDG	56
Contralateral SDG	2
Interhemispheric SDG	16
Bilateral SDG	11
Posttraumatic hydrocephalus	35/121 (28.93%)
Postoperative meningitis	2/121 (1.65%)
Posttraumatic Seizures	10/121 (8.26%)

The favorable outcome group and the unfavorable outcome group were composed of 90 (74.38%) and 31(25.62%) patients, respectively. When the two groups were compared, univariate analysis showed that an unfavorable outcome significantly correlated with the presence of Glasgow Coma Score (GCS) scores on admission, hypoxemia, pupil reactivity, the status of the basal cistern, the craniectomy site, postoperative PHI, posttraumatic cerebral infarction and PTH (all *p* < 0.05). No other statistically significant risk factors were found (Table 1).

**Figure 1:**
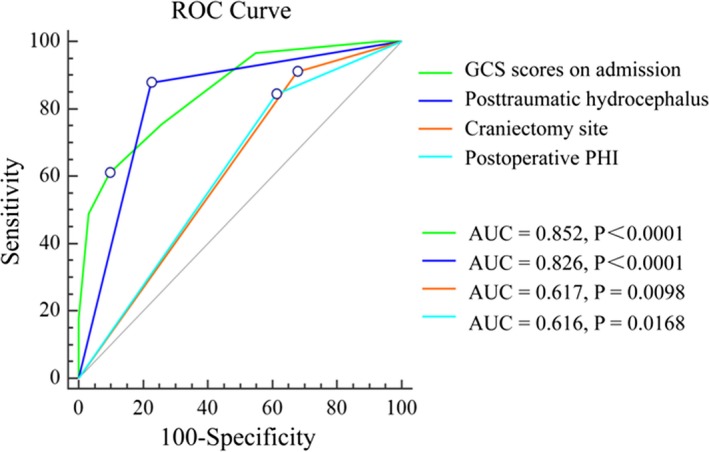
Comparison of AUC of the GCS scores on admission, posttraumatic hydrocephalus, craniectomy site, and postoperative PHI. The performance power of GCS scores on admission and posttraumatic hydrocephalus was similar to influence patient's outcome (*p* = 0.6232), and either were better predictive than craniectomy site or postoperative PHI (*p* < 0.05), respectively. (AUC: areas under the curve; GCS: Glasgow Coma Scale; PHI: progressive hemorrhagic injury; ROC: receiver operating characteristic)

Binary logistic regression analysis of the potential risk factors revealed that the presence of GCS scores on admission (OR 0.285, *p* = 0.001), PTH (OR 8.688, *p* = 0.003), the craniectomy site (OR 8.068, *p* = 0.033), and postoperative PHI (OR 6.196, *p* = 0.026) were independent risk factors that correlated with an unfavorable outcome (Table 3). Furthermore, we analyzed each of the four factors in separate ROC analyses where each variable was used individually to evaluate the power to predict the development of an unfavorable outcome after DC in TBI. As shown in Figure 1, the ROC curves demonstrated that these factors had different accuracies at predicting unfavorable outcome (area under of the curve (AUC = 0.852, 95% CI 0.776–0.910 for GCS scores on admission; AUC = 0.826, 95% CI 0.747–0.889 for PTH, AUC = 0.617, 95% CI 0.524–0.704 for craniectomy site; AUC = 0.616, 95% CI 0.523–0.703 for postoperative PHI). Notably, the performance power of the GCS scores on admission and PTH were similarly able to affect the patient's outcome (*p* = 0.623), and both of them were significantly better predictives than craniectomy site or postoperative PHI (*p < *0.05), respectively.

**Table 3:** Binary logistic regression model for factors associated with unfavorable outcome

Factors	OR	95% CI	*p* value
GCS scores on admission	0.285	0.139–0.584	0.001
Hypoxemia	2.259	0.368–13.880	0.379
Pupil reactivity	2.112	0.757–5.886	0.153
Status of basal cistern	2.729	0.555–13.408	0.216
Craniectomy site	8.068	1.182–55.087	0.033
Postoperative PHI	6.196	1.237–31.024	0.026
Posttraumatic cerebral infarction	2.639	0.361–19.309	0.339
Posttraumatic hydrocephalus	8.688	2.087–36.169	0.003

CI: confidence interval; GCS: Glasgow Coma Scale; OR: odds ratio; PHI: progressive hemorrhagic injury.

## 4 DISCUSSION

A number of factors are believed to influence the outcome of TBI patients including age (Kamal, Agrawal, & Pandey, 2016; Yuan et al., 2012), GCS scores (Khalili et al., 2016; Lemcke, Ahmadi, & Meier, 2010), pupillary reactivity (Kamal et al., 2016; Yuan et al., 2012), CT findings (Khalili et al., 2016; Yuan et al., 2012), and ICP (Khalili et al., 2016; Yuan et al., 2012). However, few studies investigated the association between postoperative complications and the long‐term outcomes of DC in adult patients with TBI as we did in this study. In the current study, we demonstrated that a low GCS score on admission, bilateral DC, postoperative PHI and PTH influenced the long‐term outcome of TBI patients. The ROC curves demonstrated that the performance power of the GCS scores on admission was similar to PTH in influencing the patient's outcomes, and both of them were better to predict the patient's outcomes than the craniectomy site or postoperative PHI.

It is generally accepted that a GCS score on admission is strongly associated with an unfavorable outcome in patients with severe TBI (Khalili et al., 2016; Leitgeb et al., 2013; Lemcke et al., 2010; Marmarou et al., 2007). A low GCS score on admission generally suggests the presence of severe brain tissue damage. The most detailed analysis of the effects of GCS scores on outcomes after severe TBI was done in the IMPACT (International Mission for Prognosis and Clinical Trial) study (Marmarou et al., 2007). The investigators found that the GCS scores at hospital admission were strongly associated with the GOS score at the follow‐up 6 months after the trauma. We could confirm the findings from this and other studies that the GCS score on admission was the most sensitive factor associated with an unfavorable outcome at the 6 months follow‐up in our study.

Our study showed that the craniectomy site (bilateral or unilateral DC) correlated with an unfavorable outcome, this effect was more pronounced in bilateral DC than in unilateral DC. Reasons for this might be that TBI patients with bilateral DC generally suffer from more severe TBI and require longer operation times with increased use of anesthetic and more frequent postoperative complications than TBI patients with unilateral DC. This suggests that the neurosurgeon needs to be careful in the use of bilateral DC in TBI patients. Moreover, large cranial defects lead to turbulence in the CSF circulation hydrodynamics and the cerebral blood perfusion due to exposure to atmospheric pressure (Manfiotto et al, 2017). Cranioplasty performed to repair large cranial defects would be helpful to correct these disturbances (Liang et al., 2007; Winkler, Stummer, Linke, Krishnan, & Tatsch, 2000). Previous studies found that an early cranioplasty was better than one later on to improve the prognosis of patients with large cranial defects after DC (Chibbaro et al., 2011; Liang et al., 2007; Nalbach, Ropper, Dunn, & Gormley, 2012). However, the optimal timing of cranioplasty after DC has not been established yet (Malcolm et al., 2016; Morton et al., 2017).

Postoperative PHI is another factor that was found to be associated with unfavorable outcome (Flint, Manley, Gean, Hemphill, & Rosenthal, 2008; Vedantam et al., 2016), and this is usually reported in the first few days following DC (Vedantam et al., 2016; Yang et al., 2008). An important reason for this may be the loss of the tamponade effect by high ICP (Kurland et al., 2015; Yang et al., 2008). PHI may lead to clinical deterioration and may require additional interventions, increasing the length of stay in hospital and placing the patient at risk for other complications, including neurological deterioration and death (Flint et al., 2008; Vedantam et al., 2016). We found that the occurrence of PHI after DC was associated with an unfavorable outcome at 6 months follow‐up. These results further emphasize the importance of preventing PHI in this population. Therefore, the prompt detection and removal of the hematoma are essential for the clinical management, and repeated routine CT is recommended after the first operation (Wen et al., 2013).

The occurrence of PTH was found to correlate significantly with unfavorable outcomes, which is consistent with previous studies (Chen et al., 2017; Honeybul & Ho, 2014). The incidence of PTH is 7.9%‐54% in patients with TBI after DC depending on the inclusion criteria and the definitions of hydrocephalus that were used (Fotakopoulos, Tsianaka, Siasios, Vagkopoulos, & Fountas, 2016; Ki et al., 2015; Kim, Lee, Ahn, Park, & Huh, 2017; Su, Lee, Huang, Su, & Chen, 2011). In our study, PTH occurred in 28.93% of the cases. PTH directly impairs the brain metabolism and often leads to reduced clinical improvement, neurological deterioration, and poorer outcomes if it is not detected and treated early on (Chen et al., 2017). However, PTH is treatable with CSF diversion by ventricular shunting, which has been reported to improve the acute clinical status, imaging findings and neurophysiologic function (Sheffler, Ito, Philip, & Sahgal, 1994; Weintraub, Gerber, & Kowalski, 2017). Therefore, PTH diagnosis and shunting early on would be useful to prevent further neurological damage in patients recovering from TBI (Yang et al., 2008).

Finally, a ROC model was created in the present study to further assess the accuracy of these factors for predicting an unfavorable outcome. Based on the AUC values, the performance power of GCS scores on admission was similar to PTH, and both of them were more likely to influence patients’ outcomes than the craniectomy site or postoperative PHI, respectively. Among the four risk factors, the GCS score on admission is commonly employed for the classification of TBI severity, which usually reflects the severity of brain injury at admission, especially the severity of primary brain injury. The implementation of craniectomy is directed by surgical indications, and the site of craniectomy is usually determined by the position of intracranial hematoma or contusion. Thus, an intervention is difficult both regarding the admission GCS scores and the site of craniectomy. In contrast, it is easier to intervene in case of postoperative PHI and PTH. The majority of postoperative PHI appear early after DC and may become lethal complications if unrecognized. Thus, an immediate postoperative diagnosis and therapeutic intervention are crucial to alleviate neurological damage induced by postoperative PHI. If the patient's condition permits, we recommend a CT scan to detect PHI immediately after DC, especially in patients have intraoperative brain swelling, postoperative neurological deterioration, pupillary dilation contralateral to the craniectomy site, and intractably elevated ICP are suggestive of PHI. PTH is easily overlooked during rehabilitation, but a treatable complication, which underlines the importance of an early diagnosis and treatment to prevent further neurological damage in patients recovering from TBI. If the patient's presence of either clinical deterioration or failure to make neurological progress over time, a CT or MRI scan is recommended to exclude PTH.

The retrospective nature of this study and a small number of patients recruited limit our ability to draw firm conclusions. Therefore, a prospective study with a large sample is needed to obtain firm conclusions.

## 5 CONCLUSION

Posttraumatic hydrocephalus and postoperative PHI were found to be independently associated with unfavorable long‐term outcome after DC in patients with TBI. Early prevention and treatment of PTH and postoperative PHI may be beneficial to improve the long‐term outcome, especially in patients with lower admission GCS scores or bilateral DC.

## CONFLICT OF INTEREST

There authors have no conflict of interest to declare.
